# Anaphylaxis: Focus on Transcription Factor Activity

**DOI:** 10.3390/ijms22094935

**Published:** 2021-05-06

**Authors:** Yanru Guo, Elizabeth Proaño-Pérez, Rosa Muñoz-Cano, Margarita Martin

**Affiliations:** 1 Biochemistry Unit, Biomedicine Department, Faculty of Medicine, University of Barcelona, 08036 Barcelona, Spain; yanruguo@ub.edu (Y.G.); ely_proano@ub.edu (E.P.-P.); 2 Clinical and Experimental Respiratory Immunoallergy (IRCE), Institut d’Investigacions Biomèdiques August Pi i Sunyer (IDIBAPS), 08036 Barcelona, Spain; rmunoz@clinic.cat; 3 Allergy Section, Pneumology Department, Hospital Clinic, University of Barcelona, 08036 Barcelona, Spain; 4 ARADyAL (Asthma, Drug Adverse Reactions and Allergy) Research Network, 28029 Madrid, Spain

**Keywords:** Mast cells, transcription factors, anaphylaxis, proinflammatory mediators

## Abstract

Anaphylaxis is a severe allergic reaction, rapid in onset, and can lead to fatal consequences if not promptly treated. The incidence of anaphylaxis has risen at an alarming rate in past decades and continues to rise. Therefore, there is a general interest in understanding the molecular mechanism that leads to an exacerbated response. The main effector cells are mast cells, commonly triggered by stimuli that involve the IgE-dependent or IgE-independent pathway. These signaling pathways converge in the release of proinflammatory mediators, such as histamine, tryptases, prostaglandins, etc., in minutes. The action and cell targets of these proinflammatory mediators are linked to the pathophysiologic consequences observed in this severe allergic reaction. While many molecules are involved in cellular regulation, the expression and regulation of transcription factors involved in the synthesis of proinflammatory mediators and secretory granule homeostasis are of special interest, due to their ability to control gene expression and change phenotype, and they may be key in the severity of the entire reaction. In this review, we will describe our current understanding of the pathophysiology of human anaphylaxis, focusing on the transcription factors’ contributions to this systemic hypersensitivity reaction. Host mutation in transcription factor expression, or deregulation of their activity in an anaphylaxis context, will be updated. So far, the risk of anaphylaxis is unpredictable thus, increasing our knowledge of the molecular mechanism that leads and regulates mast cell activity will enable us to improve our understanding of how anaphylaxis can be prevented or treated.

## 1. Anaphylaxis, a General Overview: Definition, Effector Cells and Mechanisms

Anaphylaxis, from the Greek (*ana* meaning against; *phylax* meaning guard or protection), is defined as a serious and complex allergic reaction that involves respiratory and cardiovascular symptoms and may be life-threatening specially if not treated [1]. Different triggers can cause anaphylactic reactions, the most common include drugs [2], insect venom [3], and food allergies; the last with a higher prevalence in children [4]. Sometimes anaphylaxis co-exists in patients with asthma, urticaria, mastocytosis, and atopic dermatitis (AD) [5]. Cell mechanisms involved in anaphylaxis can be distinguished depending on the presence or absence of IgE, defining an IgE-dependent or IgE-independent anaphylaxis [5,6]. The most well-defined mechanisms are described for the former type of anaphylaxis, where basophils and mast cells may be effectors, with mast cells having definitive evidence [7]. Moreover, there is a high occurrence of anaphylaxis in patients with mastocytosis, a rare pathology with increased accumulation of mast cells in one or more organs [8]. IgE-independent reaction can include: IgG-dependent anaphylaxis [6], where FcγRs signaling can elicit activation of macrophages, neutrophils, basophils, and mast cells in mice models [9], but with no direct evidence in humans; complement-dependent anaphylaxis, where C3a, C4a, and C5a peptides (also called anaphylatoxins) can activate mast cells, basophils, and macrophages, leading to the release of inflammatory mediators [10,11]. Blood levels of C3, C4a, and C5 correlate with the severity of anaphylaxis in human subjects [12]. 

Recently, MRGPRX2 dependent anaphylaxis has gained increased interest. MRGPRX2 (MAS related GPR family member X2), is a G-couple seven transmembrane domain receptor expressed abundantly in human skin mast cells [13], and reported in basophils and eosinophils [14]. This receptor has been directly linked to adverse drug reaction to neuromuscular blocking agents (NMBAs), fluoroquinolones, and antibiotics [15], [16]. Moreover, MRGPRX2 has been proposed as a biomarker for allergic asthma [17], and has also been found upregulated in chronic spontaneous urticaria (CSU) [18]. The pathophysiology related to this receptor has been updated in a review by Quan et al. [19], in this Special Issue. 

In this review, we focus on the most well-defined and commonly frequent IgE-dependent anaphylaxis in humans. This hypersensitive reaction begins with antigen recognition by IgE bound to the FcεRI receptor, which leads to FcεRI aggregation [20]. This tetrameric receptor has one α, and one β chain, and a homodimer of γ chains. The alpha chain is responsible for the IgE binding. The beta and the gamma chains contain ITAM motifs that increase FcεRI signaling. The kinase cascade that follows this activation includes MAPK, among others, and results in degranulation of preformed mediators and de novo synthesis of eicosanoids and cytokines [21,22].

## 2. Proinflammatory Mediators Involved in Anaphylaxis

The actions of the proinflammatory mediators released by sensitized cells (mast cells and basophils) after antigen exposure are responsible for the symptomatology occurring in an anaphylactic reaction [5]. These mediators are commonly divided into two categories: firstly, preformed granule products, histamine, tryptase, carboxypeptidase, chymase, heparin, chondroitin sulfate, and some cytokines; secondarily, newly formed lipid mediators, which includes platelet-activating factor, prostaglandin D2, leukotriene B4, leukotriene C4, and cytokines, and chemokines. A good correlation of these mediators with the pathophysiology of anaphylaxis is found for histamine, while the contribution of lipid mediators, such as PAF, prostaglandins and leukotrienes, in mice is evident, and to a different extent in humans [5]. Initial reaction severity has also been correlated with peak serum concentrations of IL-2, IL-6, IL-10, and TNFRI [23].

Histamine is a vasoactive amine synthesized by histidine decarboxylase (HDC), which catalyzes the decarboxylation of histidine. Histamine is the prototypic mediator of anaphylaxis in humans and mice; it is mainly released by mast cells and basophils, and its systemic release causes transient hemodynamic changes, systemic hypotension, and airway obstruction. Histamine blood levels correlate with the severity and persistence of symptoms [24]. These effects derive from its binding to the H1 and H4 receptors [25]. 

Tryptases are serine proteases and the major components of mast cell granules (to a lesser amount in basophils). The total serum level of tryptase is the gold standard for diagnosis of anaphylaxis, correlating with the severity of the reaction, and they help as mediators of some of the clinical symptoms of anaphylaxis, such as urticaria, angioedema, and bronchospasm [12,24]. Four genes encode the human mast cell tryptases: *TPSG1, TPSB2*, *TPSAB1,* and *TPSD1*. The first (γ tryptase) encodes the only membrane bound member of the family, the rest are soluble tryptases. *TPSB2* encodes for βII and βIII tryptases, *TPSAB1* encodes for α and βI and *TPSD1* for δ-tryptase. Only α and β are likely to contribute to circulating tryptase levels, with β being the principal active tryptase in anaphylaxis [26].

The murine counterparts of these genes are *Tpsg1*, *Tpsb2,* and *Tpsab1* (no expression of TPSD1 is found in mice), encoding mTMT, mMCP6, and mMCP7, respectively. The first is transmembrane and the rest soluble [27]. 

Additional diversity, in both species, is achieved via allelic variation. It was recently reported in humans that an increased risk for severe anaphylaxis is associated with inherited differences in number of copies of *TPSAB1* that encodes α-tryptase [28].

Elevation of tryptase in blood is not seen in all cases of anaphylaxis, some reports suggest that this may depend on the mast cell subtype (mucosal mast cells tend to have less tryptase per cell than skin mast cells) or antigen administration (gut anaphylaxis tryptase levels may end up in the gut rather than the bloodstream) [26].

Platelet activating factor (PAF) is a proinflammatory phospholipid derived-mediator synthesized by mast cells, basophils, platelets, and other immune cells. It is negatively regulated by PAF-acetylhydrolase (PAF-AH), which hydrolyses PAF to an inactive compound. Although not within the range of histamine or tryptase, the levels of both are inversely associated with severe anaphylaxis [12,24]. Deficiency of PAF-AH predisposed patients to severe anaphylaxis [29]. In mouse models, injection of PAF induces anaphylaxis that can be blocked to a different extent with a PAF antagonist [30].

### 2.1. Cysteinyl Leukotrienes (CysLTs) and Prostaglandins

CysLTs (leukotriene C4 (LTC4), leukotriene D4 (LTD4), leukotriene E4 (LTE4)), and prostaglandins are synthesized from arachidonic acid by a variety of cells, including mast cells. Urine metabolites LTE4, and 9α,11β-PGF2 from degradation of CysLTs and PGD2 respectively, are increased in human anaphylaxis. Although they can play a role as a biomarker, their contribution to the pathophysiology of anaphylaxis remains uncertain [31].

### 2.2. Cytokines and Chemokines

The blood concentration of cytokines and chemokines is delayed in human anaphylaxis, consistent with the fact that they are mostly newly synthesized products. Several cytokines might contribute to the pathophysiology of anaphylaxis [5]. Cytokines IL-4, IL-5, and IL-13 are produced by Th2 cells and play a role in IgE production, amplifying the response to vasoactive mediators; proinflammatory cytokines IL-1β, IL-6, TNFα, and TNFRI help magnify responses in mast cells and other immune cells; conversely, IL-10 plays a negative role, attenuating responses. However, only IL-6, IL-10, and TNFRI have been found consistently increased and can be associated with the severity of anaphylaxis [12,23]. Regarding chemokines, the CCL2 chemokine is increased in human anaphylaxis, thus its signaling cascade might be important for basophil chemotactic activity during anaphylactic reactions [32]. It was previously defined that, in the absence of allergen, histone deacetylase 3 binds to the promoter sequences of CCL2 to suppress CCL2 expression. Upon allergen stimulation, histone deacetylase 3 binds to FcεRI, and mediates passive anaphylaxis by increasing the expression of CCL2 in mast cells [33].

## 3. Transcriptions Factors Involved in Anaphylaxis

The transcription factors (TFs) involved in anaphylaxis can range from TFs involved in mast cell phenotype maintenance, synthesis of proinflammatory mediators and secretory granule homeostasis to TFs involved in the determination of Th1/Th2 balance [34,35,36,37]. The shift towards Th2 determines allergic response, while Th1 cytokines are supposed to suppress these reactions [38,39]. The differential expression or regulation of TFs is involved in the basis of the mechanism concerning mast cell activity and the magnitude of responses. Consequently, to delve into TF action may allow us to better understand allergic responses, particularly the severity of these reactions. This review will focus on TFs involved, directly or indirectly, in mast cell or basophil activity in the context of anaphylaxis. Since data in humans are limited, given the life-threatening nature of anaphylaxis and ethical concerns, we added some studies performed in mice models.

### 3.1. The GATA Family

GATA transcription factors are a family of TFs (GATA1, GATA2, GATA3, GATA4, GATA5, and GATA6), characterized by their ability to bind to the DNA consensus sequence (T/A) GATA(A/G). GATA1, GATA2, and GATA3 play roles predominantly within the hematopoietic system [40,41]. GATA 4, GATA5, and GATA 6 are expressed in non-hematopoietic tissues, such as cardiac, gastrointestinal, endocrine, and gonadal system [42]. GATA1 and GATA2 are abundantly expressed in mast cells [43]. 

GATA1 null mice die at the embryonic stage due to severe anemia [44]. GATA1 knockdown mice (with intact coding sequence but lacking the promoter or with genetic modifications in critical regulatory sequences) proved GATA1 to be critical for mast cell differentiation and later stages of mast cell development in vivo [45,46] and in vitro [47,48]. More recently, studies performed with tamoxifen-inducible GATA1 knockout mice with a complete ablation of GATA1, have shown dispensable for mast cell differentiation in adult mice [49]. Although mast cell specific genes, such as *KIT* and *FcεRIa* are sustained in GATA1 deficient bone marrow derived-mast cells (BMMCs), levels of mast cell proteases were uniformly reduced, especially mast cell tryptases *Tpsg1* (mTMT), *Tpsb2* (mMCP6), and *Tpsab1* (mMCP7), suggesting that GATA1 might play a role in more specific functions of mast cells by regulating tryptase genes [49]. Aside from mast cells, GATA1 plays an important role in basophil function. Impaired degranulation and IL-4 production upon allergen-mediated activation is found in GATA1-knockdown basophils. Moreover, ΔdblGATA mice generated by deleting a high affinity double GATA site in the Gata1 promoter region, present basophilopenia, and aberrant basophil function [50].

GATA2 is essential for the differentiation of pre-basophil and mast cell progenitors (pre-BMPs) into basophils and mast cells [36]. Aside from that, GATA2 is also crucial to maintain the characteristics of mast cells. The lack of the GATA2 DNA domain can dedifferentiate mast cells into myeloid cells (macrophage and neutrophil-like) after appropriate cytokine addition [51]. Studies performed in human primary mast cells and LAD2 using siRNAS against GATA1 and GATA2, as well as ChIP assay data, show that both transcription factors are involved in FcεRIα transcription via recruitment to the *FCER1A* promoter, whereas GATA2 positively regulates the *MS4A2* promotor (encoding FcεRIβ). Suppression of these TFs leads to downregulation of FcεRI expression and IgE-mediated degranulation activity in human mast cells [52]. Furthermore, GATA2 deficiency in human subjects show decreased expression of KIT and FcεRI on mast cells and, consequently, IgE dependent degranulation is impaired [53].

Assessment of protease transcription regulation has been performed in mouse BMMCs using siRNas from GATA1 and GATA2, as well as combination of both. The results show that GATA2 knockdown suppresses a wider array of mast cell proteases than GATA1. Indeed, *Cpa3, Mcpt4, Mcpt8* and *Cma1* are significantly downregulated after GATA2 silencing, but not in GATA1 knockdown cells [27]. GATA2 has a prominent role regulating the mouse tryptase genes *Tpsg1* (mMTM), *Tpsb2* (mMCP6), but coordinately works with GATA1 facilitating each other’s DNA binding activity to upstream regions of these genes [27]. These results are similarly found in GATA2 deleted-DNA domain in mice BMMCs [51]. GATA2 also affects *Mcpt1* and *Mcpt2* gene expression, which are specific proteases in mucosal mouse mast cells [54]. 

GATA3 is generally essential for T cell differentiation from the earliest stages as an effector molecule in Notch signaling [55,56], and it is considered a master regulator of T helper type 2 differentiation [57]. Unexpectedly, overexpression of GATA3, in the absence of Notch signaling, can activate the mast cell-like program (upregulation of c-KIT, GATA1, GATA2, MITF, CPA3, and the effector tryptase, mMCP6) without mast-cell growth factors IL-3 and SCF [58]. It was recently reported that GATA3 is expressed in a newly described population of T follicular helper cells (Tfh13), tasked with the production of high-affinity anaphylactic IgE, but not for low-affinity IgE. Tfh13 expressed IL-4, IL-5, and IL-13, and was found in patients allergic to aeroallergens or peanuts [59]. Helminth infection elicits a Tfh2 response leading to IgE switching, resulting in low-affinity IgE antibodies. These Tfh2 cells do not express GATA3 and cannot make IL-13. Conversely, allergens induce GATA3^+^Tfh13 cells driving high-affinity IgE production and anaphylaxis. Previously, it was reported that IL-13 works synergistically with IL-4 to promote high affinity IgE [60,61]. Consistent with these data, IL-13 has been genetically associated with elevated IgE, food allergy and asthma [62,63,64], and loss of IL-13 does not impair low affinity IgE responses, such as parasitic infection [65]. Detection of the Tfh13 population in peripheral blood from allergic patients may be a promising and useful tool that will deserve future study [66]. Moreover, inhibition of IL-13 or GATA3 may be therapeutic targets for anaphylaxis. In this regards, inhibition of GATA3 activity with GATA3 DNAzyme has shown efficacy in asthmatics [67]. In addition, deacetylation of GATA3 leads to suppression of the Th2 immune responses in asthma [68]. 

### 3.2. The STAT Family

The transducer and activator of transcription (STAT) family has seven members including STAT1, STAT2, STAT3, STAT4, STAT5 (STAT5A and STAT5B), STAT6. Four known JAK proteins can activate one or more of the STAT members. The JAK-STATS signaling pathway is downstream of several cytokines and growth factor receptors. Inhibition of JAKs abrogates mast cell activity [69]. Indeed, inhibition of JAK 1 and 2 with ruxolitinib was reported to remit food allergy in mice, mainly through immunosuppression and the prevention of mast cell hyperplasia, and partially through the inhibition of mast cell activation [70]. Indeed, ruxolitinib has been reported to diminish mast cell degranulation and cytokine synthesis in vitro [71], and to ameliorate symptoms in aggressive mastocytosis [72]. 

STAT1 was reported to enhance secretion of Il-13 and expression of IL-13Rα 1 in human mast cells [73].

STAT3, aside from its transcriptional activity, has a non-canonical role modulating mitochondrial activities enhancing ATP production required for mast cell exocytosis events. Short inhibition of STAT3 significantly decreases mouse and human mast cell degranulation, as well as passive systemic anaphylaxis [74]. Interestingly, the endogenous inhibitor PIAS translocates to the mitochondria after mast cell activation, inhibiting STAT3 and regulating the process. In consequence, PIAS silencing leads to enhanced cell degranulation indicating that it could be a target for controlling the extent of mast cell degranulation [74]. Mitochondria STAT3 inhibitors, developed with no effect on STAT3 transcriptional activity, may be potential therapeutic agents for mast-related diseases [75].

On the other hand, loss or inhibition of STAT3 in rodents resulted in an increase in stability and expression of vascular endothelial protein (VE)-cadherin/βcatenin complexes that leads to a reduction in vascular permeability and inhibits anaphylaxis [76], suggesting an involvement of STAT3 in gap junction integrity in endothelial cells. The inhibition of STAT3 regulating mast cell degranulation is clearly diminished in human mast cells, but not in rodent mast cells, indicating that STAT3 inhibition may involve various mechanisms. Some authors found that dominant-negative STAT3 mutations in hyper IgE syndrome patients (AD-HIES) show diminished food allergies and anaphylaxis compared with other hyper-IgE patients with non-mutated STAT3 [77], reinforcing the idea that suppression of STAT3 activity ameliorates allergic symptoms and the incidence of anaphylaxis. 

STATs 1 and 3 play a role downstream to IFN I signaling. IFN is used in the treatment of mastocytosis [78,79,80]. Steady state IFN I exposure appears to confine mast cells to less mature states and to limit the onset of anaphylactic responses. Blockage of IFNI signaling increases the severity of IgE-dependent anaphylaxis [81]. Consequently, IFNI receptor knockout mice show an increase in anaphylaxis and histamine release. Analysis of these deficient mice show the same number of mast cells and FC ε RI expression, but an increase in secretory granule synthesis and exocytosis. According to this, the level of TFEB, a transcription factor regulator of granule biogenesis in mast cells [82], is enhanced. Biogenesis of secretory granules in mast cells and the dynamics of exocytosis deserve being considered a plausible cause underlying the severity of anaphylaxis. On the other hand, STAT3 inhibition in the background of STAT1 deficiency resembles the IFNR knockout mouse phenotype, showing an increase in TFEB, secretory granule synthesis, exocytosis and systemic anaphylaxis [81]. Consistent with this, STAT3 has been found associated with TFEB in the nucleus, partly suppressing its function [83]. 

Altogether, loss of STAT3 activity has been found to reduce the incidence of anaphylaxis in humans; however, STAT3 also plays a role downstream to IFNI signaling, limiting severity of anaphylaxis in mouse models. The model analyzed, the different nature of the inhibitors used, and the specific signaling receptor context may be the basis of these discrepancies that deserve further consideration.

STAT4 is only expressed in connective-tissue mast cells (CTMCs) [84] and suppresses the proliferation of CTMCs via IL-6 regulating KIT ligand in an autocrine mechanism [85].

STAT5 plays a critical role in basophil and mast cell differentiation and maintenance via direct binding to the promoter and an intronic region of the *GATA2* gene [86]. Overexpression of GATA2 is sufficient to drive the differentiation and maintenance of these types of cells [86]. STAT5 is found downstream of KIT signaling, consequently providing critical mast cell growth and survival functions. Indeed, STAT5A/B deficient mice lack tissue mast cells in vivo [87], and the phenotype resembles mice with mutations in KIT or its ligand, Stem cell factor (SCF) [88]. STAT5 lies downstream of oncogenic D816V KIT, a hall mark mutation in mastocytosis [89], so it could be a therapeutic target for drug resistance to systemic mastocytosis [90]. STAT5 could also be a therapeutic target for the treatment of chronic inflammatory skin disease, such as atopic dermatitis, where increased mast cell number with high levels of phospho-STAT5 were found in skin lesions from some patients [91]. 

BMMCs can be obtained from STAT5 knockout mice if maintained under IL-3 plus stem cell factor (SCF) conditions, which do not appear to exist in vivo [92]. These results indicate that STAT5 expression is not required for normal mast cell development but is necessary to support subsequent survival and proliferation. Moreover, the same authors show that STAT5 is important for murine BMMCs, IgE degranulation, leukotriene B4 production and IL-13, IL-4, and TNF alpha cytokine synthesis. Interestingly, STAT5 delivers signals for cell survival and increased cytokine mRNA stability rather than transcription, repressing the mRNA-destabilizing protein tristetraprolin (TTP) [92]. Although the STAT5 mechanisms in IgE degranulation and leukotriene B4 production in mast cells are unclear, it has been reported that STAT5 co-localizes with FcεRI upon receptor crosslinking being STAT5 activation dependent on Fyn kinase [93].

Furthermore, STAT5 has been reported acting downstream of the MRGPRX2 receptor after thymic stromal lymphopoietin (TSLP) stimulation indicating a role in atopic skin diseases via this receptor [94], and providing insights into MRGPRX2 signaling.

STAT6 is required for class switch recombination to IgG1 and IgE and type 2 immune responses against helminths or allergens [95]. IgE-dependent degranulation has been explored in STAT6 deficient mice, showing a reduction in the late phase of allergic reaction, cytokine production (IL-6, TNF alpha) rather than in the early phase. No differences were found in histamine and leukotriene C4 [96]. Genetic variation in the *STAT6* gene may be associated with predisposition to allergic diseases [97,98].

### 3.3. The MiTF/TFE Family 

The MiTF/TFE family of basic helix-loop-helix leucine zipper (b-HLH-LZ) transcription factors includes MITF, TFEB, TFE3, and TFEC. The b-HLH-LZ recognizes the E-box (CANNTG) motifs in the promoter region of target genes [99]. MITF is mainly expressed in melanocytes, mast cells, osteoclasts, macrophages, NK cells, B cells, and heart; while TFEC expression is restricted to cells of myeloid origin [100]. The other members, TFE3 and TFEB, show a wider pattern of expression [101,102].

The microphthalmia-associated transcription factor (MITF) is involved in the generation and function of mast cells [103], melanocytes [104], osteoclast [105,106], and retinal pigmented epithelium [107]. There are several isoforms described, MITF-A, -B, -C, -D, -E, -H, -M, Mc, and -J, differing in their exon 1 and sharing common downstream exons from 2 to 9 [108]. MITF is essential in mice for the differentiation of mast cells from bone marrow hematopoietic stem cells to tissue [109]. MITF lies downstream of the STAT5 signaling required for the differentiation of pre-basophil and mast cell progenitors into mast cells, but not in basophils. Instead, C/EBPα is a critical transcription factor for basophil cell fate in mice [110]. Therefore, there is an antagonist regulation between MITF and C/EBPα, specifying mast cell and basophil fate respectively. The most widely expressed isoform, MITF-A, as well as the more restricted MITF-MC and MITF-E, have been described in mucosal type mast cells (from primary BMMCs to intestinal mast cells). BMMCs from Mitf^−/−^ mice show hypogranularity and defective SCF dependent migration that can be restored with any of the mast cell isoforms described. These isoforms regulate a common transcriptome that involves chymases (mMCP1, mMCP2, mMCP4, mMCP5), tryptase (mMCP6), granzyme B, and adhesion molecules, but also regulate a unique set of genes. Cathepsin G, α4integrin, and mMCP8 may be preferentially regulated by MITF-MC and MITF-E, while KIT expression would be regulated by MITF-A [111]. MITF-A is strongly expressed in both human CD34^+^ progenitor derived mast cells (hMCs) and HMC-1 cells, and regulates the level of tryptase-β1 [112]. 

The *Mitf* mutant mouse is strikingly similar to SCF or Kit-deficient mice as regards mast cells and melanocytes [88,99,113]. MITF is highly expressed in mastocytosis. Indeed, MITF expression is regulated by KIT-dependent signals and is required for the transformed phenotype of mastocytosis [114]. Normal KIT signaling, as well as oncogenic signaling, can regulate the expression of MITF at posttranscriptional levels via miR-539 and miR-381 [115].

It was recently shown that the GATA2–MITF axis is critical for IgE/mast cell–mediated anaphylaxis. This study shows GATA2 as a primary transcription factor, followed by MITF, for controlling histamine synthesis via the transcription of histidine decarboxylase (HDC). Indeed, overexpression of MITF in the absence of the *GATA2* gene can fully restore the *c-KIT* gene, and partial restore the *HDC* gene [116]. These data support that GATA2, in addition to inducing MITF expression, would maintain the accessibility of MITF to the *HDC* promoter. Moreover, MITF plays a key role in lipid mediators on mast cells, increasing expression of PGD2 [117].

MITF activity is repressed by the histidine triad protein (HINT1) in quiescent mast cells. Upon IgE crosslinking, there is an increase of diadenosine tetraphosphate (Ap4A) in the nuclei, which binds to HINT1 but not to MITF, dissociating the MITF/HINT complex, generating MITF free to bind to target genes and induce their transcription [118]. A recent study highlighted the length of the phosphodiester linkage of Ap4A with the ability to dissociate the MITF/HINT complex [119].

The transcription factor EB (TEFB), member of the MiTF/TFE family, as mentioned in the STAT paragraph above, is critical for secretory granule biogenesis in mast cells [81]. TFEB phosphorylated by mTORC1 is retained outside the nucleus by associating with 14-3-3 proteins. Reduction in mTORC1 level leads to TFEB translocation to the nuclei, regulating transcription of genes involved in granule content and biogenesis. TFEB dysregulation alters secretory-granule conditions, disturbing mast-cell secretory functions and the IL-33 signaling pathway [82].

**Figure 1:**
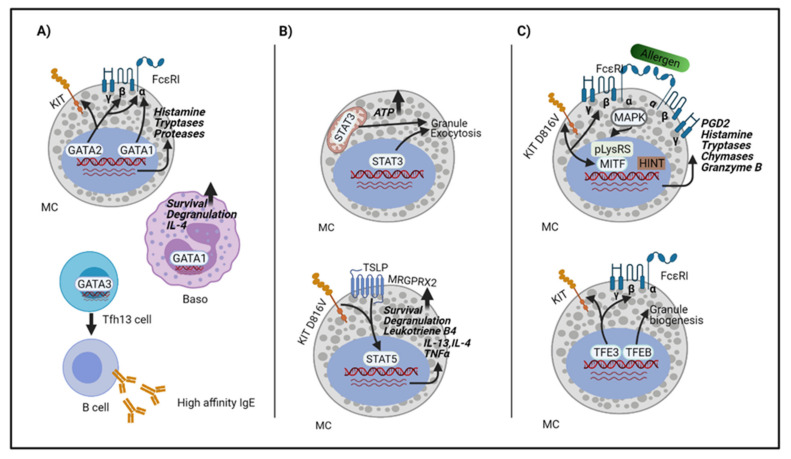
GATA, STAT, and MITF family in anaphylaxis. (**A**) GATA family plays a central role in mast cell activity. GATA2 is key for mast cell activity. FcεRIα, FcεRIβ, and KIT expression is regulated by GATA2, while FcεRIα is also controlled by GATA1 [52,53]. GATA1 regulates Il-4 production in basophils [50]. GATA1 and GATA2 regulate tryptases (including mTMT, mMCP6, and mMCP7). Furthermore, GATA2 regulates histamine and proteases (Cpa3, Mcpt4, Mcpt8, Cma1) synthesis [27,54]. GATA3 defines a Tfh13 population involved in anaphylactic IgE production [59]. (**B**) STAT family is downstream of key receptors and controls mast cell activity. STAT3 increases IgE-dependent degranulation in a canonical and non-canonical pathways increasing ATP production in mitochondria [74]. STAT5 is downstream of KIT D816V in mastocytosis, it increases cell survival, degranulation, leukotriene synthesis and stabilizes IL-13, IL-4, and TNFα mRNA [92,93]. Thymic stromal lymphopoietin (TSLP) binding to MRGPRX2 increases STAT5 activity [94]. (**C**) IgE-LysRS-MITF axis in anaphylaxis. MITF is downstream of KIT and IgE signaling [114,118]. MITF and KIT are reciprocally regulated [114,115]. Upon IgE-Ag, LysRS is phosphorylated by the MAP kinase pathway and translocated into the nucleus. LysRS activity in the nucleus dissociated MITF–HINT complex, inducing MITF transcriptional activities [118]. TFE3 is important for IgE–dependent pathway anaphylaxis by regulating FcεRI and KIT receptor expression [120]. TFEB is a master transcription factor for mast cell granule [81,82].

The transcription factor E3 (TFE3) is very close to MITF, however it seems not to play a role in mast cell development, but in mast cell activation. Tfe3 knock out mice show similar mast cell numbers; however the levels of KIT and FcεRI expression were reduced in peritoneal and cultured mast cells and, consequently, cell degranulation and mediator release were limited [120] (Figure 1).

### 3.4. Other Transcription Factors

The CCAAT-enhancer-binding protein (C/EBP) transcription factor alpha (C/EBPα) plays an important role in the differentiation of basophils from pre-BMP [110]. C/EBPα expression is low in basophil/mast cell progenitors (BMCPs) and its expression is upregulated in basophils, but downregulated in mast cells during cell differentiation, indicating that the level of C/EBPα expression plays an antagonist role in basophil and mast cell lineage commitment [110]. Moreover, GATA2 directly binds to the *C/EBPα* gene inhibiting its expression; concomitantly the loss of GATA2 activity by DNA binding depletion (GATAΔCF) enhances C/EBPα expression. Altogether this indicates that GATA2 maintains mast cell identity, downregulating C/EBPα expression [51]. Experiments reducing or overexpressing C/EBPα levels in committed mast cell show increase and suppression of granule formation, respectively. In agreement with this, Mcpt1 and Mcpt2 levels were increased in C/EBPα knockdown and reduced in C/EBPα overexpression, MITF levels being unaffected. Interestingly, in all cases, mast cell degranulation remains similar to control cells [121]. 

Transcription factor PU.1 is a hematopoietic cell-specific transcription factor, which plays a role in mast cell development and function cooperatively with GATA1 and GATA2. PU.1 is involved in FcεRI expression by transactivation of FER1A (encoding FcεRI α) on human mast cells, consequently, PU.1 silenced human mast cells have impaired IgE-mediated degranulation [52]. More recently, the same authors performed in vitro and in vivo PU.1 knockdown experiments in mouse mast cells. In this case, PU.1 knockdown reduces Syk and FcεRI β expression and consistently decreases allergic inflammation [122]. PU.1 knockdown has been reported to reduce IL-33 signaling (driver of Th2 responses) in human mast cells and basophils [123]. Altogether, knockdown of PU.1 would suppress mast cell activation.

### 3.5. Transcription Factors in Mast Cell Activation

The above family of transcription factors play a critical role in regulating mast cell development, and in the identity and function of mast cells. Apart from this, the transcriptional machinery is activated after IgE-Ag and SCF engagement via the FcεRI and KIT mast cell receptors respectively [124]. NF-kB, NFAT, and AP-1 TFs are predominantly involved in the acute regulation of inflammatory genes [125,126,127,128]. Early growth response 1 (EGFR1) and EGFR2, members of the zinc finger family, have been shown to regulate TNF and IL-13 production after IgE-Ag stimulation and SCF engagement [129,130]. SCF-mediated mast cell activation was also shown to promote cytokine production through activation of MAPKs, including TFs NF-kB and NFAT [131,132].

MITF activity is enhanced after IgE-Ag stimulation in mast cells. An important mediator of this pathway is Lysyl-tRNA synthetase (LysRS), a moonlight protein with an essential function in translation (catalyzes the binding of lysine to its cognate tRNA), and a non-canonical function in mast cells. Upon IgE activation, LysRS becomes phosphorylated by MAP kinases at serine 207, this phosphorylation leads to conformational changes resulting in the translocation of the protein to the nucleus and the switching of its catalytic properties, now synthesizing Ap4A, a dinucleotide that releases MITF from HINT, allowing MITF-dependent gene transcription [118,133,134]. 

## 4. Host Genetic Factors or Mutations Related to Anaphylaxis 

Multiple factors may predispose people to anaphylaxis, apart from environmental causes difficult to predict, genetic modifications can be key. Polymorphisms, as well as mutations, that can influence anaphylaxis have been investigated in order to understand the molecular basis and to search for biomarkers and therapeutic tools.

Genetic analysis of cytokines playing a key role in Th2 and IgE switching have been assessed in order to find differences that could correlate with the severity of allergic reactions. Genetic polymorphisms (SNPs) of the Il-4 Rα gene (involved in signaling transduction of IL-4 and IL-13) and IL-10 have been associated with drug allergy in atopic women [135]. In mice infected with a nematode parasite, IL-4/IL-13 production and IL-4R-alpha signaling are enhanced, thereby increasing vascular permeability and exacerbating anaphylaxis [136]. In that context, a polymorphism in STAT6, a transcription factor involved in both IL-4 and IL-13 production, has been associated with allergic diseases [97]. A pathologic role for IL-18 includes stimulating mast cell and basophil degranulation, recruiting immune cells, promoting type II T helper cell (Th2) response and IgE switching. Although, IL-18 polymorphisms have been described in relation to allergic diseases, no substantial evidence covering anaphylaxis has been found [137].

Altogether, to our knowledge a direct correlation of cytokine polymorphisms with anaphylaxis has not been clearly found in humans. 

Among the gene mutations described that might correlate with anaphylaxis severity, notably, the D816V activating mutation in KIT is the most consistent, confirmed by recent data from the European Anaphylaxis Registry [138]. In more than 95% of patients, the KIT activating mutation D816V is identified as the underlying cause of mastocytosis, the clonal expansion of mast cells is found as the basis of the onset and severity of allergic reactions [139]. Recently, the aberrant KIT D816V signal was associated with enhanced STAT5 phosphorylation and activity that contributes to an increase in IL-6 serum levels and a higher percentage of IL-6 positive mast cells, which associates with disease severity and progression [89]. This aberrant KIT signal has also been reported to activate NFAT constitutively [140]. 

As described above, heterozygous loss of function of GATA2 mutations in human subjects was also reported to limit IgE-dependent degranulation in human mast cells and to reduce IgE-mediated clinical allergic disease. These findings are antigen dependent and associated with reduced KIT and FcεRI expression. Interestingly, a numeric problem in mast cells or an impaired response to the mast cell mediator histamine was not found [53] 

The loss-of-function mutations in STAT3, found in autosomal dominant-hyper IgE syndrome (AD-HIES) correlates with lower rates of anaphylaxis, which may be explained by reducing mast cell degranulation [77], and affecting the architecture and functional dynamics of endothelial junctions, thus preventing vascular permeability [76].

**Figure 2:**
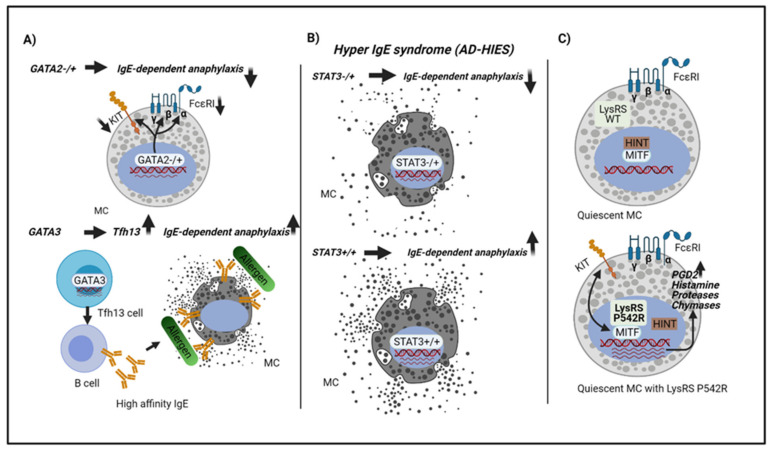
Host factors or mutations related to transcription factors in anaphylaxis. (**A**) Loss of GATA2 function in human subjects result in lower KIT and FcεRI expression and IgE-dependent degranulation impaired [53]. GATA3 is essential for Tfh13 cell production, which regulates B cells to produce anaphylactic IgE [59]. (**B**) Loss of STAT3 function in autosomal dominant-hyper IgE syndrome (AD-HIES) shows a decrease in anaphylaxis compared to normal STAT3 activity [77].(**C**) LysRS P542R induces a conformational change in LysRS allowing translocation into the nucleus, activating MITF, and relative targets genes in the absence of stimuli (quiescent cells). Eventually, the allergen encounter leads to anaphylaxis [141].

More recently, our group found a mutation in the *KARS* gene that encodes Lysyl-tRNA synthetase (LysRS) associated with severe anaphylaxis to wasp venom [141]. As discussed in the above paragraph, LysRS increases MITF activity upon IgE-Ag activation in mast cells [118]. We found that mutation P542R LysRS (proline replaced by arginine at amino acid 542) results in conformational changes and translocation of the mutant protein to the nucleus. The structural analysis shows the mutant in an open state that resembles the phosphorylated serine 207 LysRS activated form. Interestingly, this mutation enhances MITF activity and increases transcription of target genes, such as *Hdc* and *Cma* (Chymase), in quiescent mast cells. Upon IgE activation, this mutation enhances mast cell degranulation and PGD2 synthesis and release [141] (Figure 2). 

## 5. Conclusions and Perspectives

Anaphylaxis is a serious allergic reaction that may be life threatening and seriously affects the quality of life of patients. Thus, there is a clear need to identify biomarkers and therapeutic targets for identification and treatment. The exacerbated release of mediators by mast cells and basophils is the main cause of anaphylaxis. Although the causes of this high aberrant response can be multiple, alteration in transcriptional activity of proinflammatory mediators and mast cell granule homeostasis may contribute to the severity of the reaction. 

Transcription factors that target receptors or mediators involved in anaphylaxis has been summarized (Table 1). Alterations in FcεRI expression by loss of function of transcription activity such as GATA2 or PU.1 can limit mast cell response and may be targets to reduce effector cells activity. GATA3 and IL-13 may also be targets to downregulate the Tfh13 population, involved in anaphylactic IgE production. Interestingly, the identification and characterization of Tfh13 presence in patients may work as biomarker that might help to stratify the severity of reaction. Histamine levels are the main cause responsible for the physiopathology of anaphylaxis. Given that MITF activity is involved in histamine synthesis and granule biogenesis, exploring the IgE-LysRS-MITF axis could shed some light on the mechanisms underlying the severity of the anaphylaxis reaction. In conclusion, modulation of specific transcription factor expression using therapeutic siRNA or specific DNAzyme, although challenging, may represent clinical benefits if off-target or non-desirable effects can be overcome.

## Figures and Tables

**Table 1:** Transcription factors relevant to IgE-dependent anaphylaxis and their targets or mechanisms described.

TF	Target or Action Related to Anaphylaxis	Cells	References
GATA1	FcɛRI αchain Tryptases IL-4	MC Basophils	[27,47,48,49,50,52]
GATA2	FcɛRI α/ β chain Tryptases Histamine Proteases	MC Basophils	[27,51,52,53,54]
GATA3	Anaphylactic IgE synthesis IL-13, IL-4, IL-5	Thf13 Th2	[57,59,60,61,66]
STAT1	IL-13Rα1	MC	[73]
STAT3	Increase degranulation	MC	[74,77]
STAT4	IL-6	MC	[84,85]
STAT5	Increase degranulation Leukotriene synthesis IL-4, IL-13 and TNFα mRNA stabilization	MC	[92,93]
STAT6	IL-6 TNFα	MC	[96,97,98]
MITF	Histamine Chymases Tryptases Granzyme B PGD2	MC	[111,112,116,117]
TFE3	FcɛRI KIT	MC	[120]
TFEB	Granule Biogenesis	MC	[81,82]
PU.1	FcɛRI IL33	MC Basophils	[52,122,123]
